# Corneal thickness, epithelial thickness and axial length differences in normal and high myopia

**DOI:** 10.1186/s12886-015-0039-6

**Published:** 2015-05-07

**Authors:** Xiaogang Wang, Jing Dong, Qiang Wu

**Affiliations:** Shanxi Eye Hospital, Shanxi, P.R. China; The First Hospital of Shanxi Medical University, Shanxi, P.R. China; Affiliated Sixth People’s Hospital Shanghai Jiao Tong University, Shanghai, P.R. China

**Keywords:** Axial length, Corneal thickness, Epithelial thickness, High myopia, Optical coherence tomography

## Abstract

**Background:**

Corneal biometric parameters can possibly be influenced by high myopia (HM). The influence of HM on corneal thickness (CT), epithelial thickness (ET) has not yet been clearly established. The aim of this study is to observe ET, CT and axial length (AL) differences between in normal and subjects with HMs and to investigate factors influencing the corneal biometric parameters and AL, such as age and gender.

**Methods:**

A total of 97 normal subjects (97 eyes) and 48 HM subjects (48 eyes) were included. The ET and CT of the central 6-mm diameter (17 regions) and the AL data were captured. The 17 corneal and epithelial regions were the center (1 mm radius, area a), the inner ring (2.5 mm radius, area b), the outer ring (3 mm radius, area c) and the 8 radial scan lines in eight directions (Superior (1) , SN (2), Nasal (3), IN (4), Inferior (5), IT (6), Temporal (7), ST (8)) with an angle of 45° between each consecutive scan line (a, b 1–8, c 1–8).

**Results:**

The ALs were increased about 4 mm in the HMs (P < 0.001). No differences in ET were observed; in contrast, significantly thicker CTs were observed in the HMs in 16 regions except the b5 subregion. In normal group, age was negatively correlated with AL but not CCT and CET and gender was correlated with CET. In HM group, age was not correlated with CCT , AL or CET and gender was correlated with AL and CCT but not CET.

**Conclusions:**

CT was thicker in the HMs but not ET. Age and gender should be considered for AL, CT and ET in both normal and HM group.

## Background

Corneal biometric parameters and axial length (AL) can possibly be influenced by the degrees of refractive error. The influence of refractive error on corneal thickness (CT), epithelial thickness (ET) has not yet been clearly established [1-3]. High myopia (HM) plays an important role in visual impairment and is highly common in China, particularly in the school population [4,5]. HM patients are at greater risks of posterior subcapsular cataracts, glaucoma and chorioretinal abnormalities [6]. The known severe visual problems primarily result from posterior segment complications, such as macular holes, choroidal neovascularization, retinal detachment, retinoschisis, etc. Regarding the anterior segment, HM may affect CT in different age groups [1,2]. However, little research into comparing mid-peripheral CT and ET between normal subjects and those with HM on Chinese populations. Moreover, the central CT (CCT) results in HM is controversial and this require further study to clarify the topic [1,2,7].

Therefore, the purpose of this study was to evaluate CT, ET and AL differences between Chinese normal and HM groups to clarify the changing tendency of central, mid-peripheral CT, ET in Chinese HMs.

## Methods

This study was performed at the Affiliated Sixth People's Hospital *Shanghai Jiao Tong University* (Shanghai, China). The research protocols were approved by the institutional review boards of the Affiliated Sixth People's Hospital *Shanghai Jiao Tong University* (Shanghai, China) and performed in accordance with the tenets of the Declaration of Helsinki. Written informed consent was obtained from each subject after they were provided with an explanation of the nature of the study.

### Subjects

We just chose Han Chinese subjects to eliminate the possible influences of different ethnic groups. The normal and HM subjects were chosen from the Ophthalmic Clinic Center at the Shanghai Sixth People’s Hospital. One random eye of each subject was chosen for this study. The inclusion criteria for the normal subjects included the following: a best-corrected visual acuity (BCVA) of ≥ 16/20, a refractive error < 5 diopter (D) spheres, normal slit-lamp and fundoscopy examinations, an IOP < 22 mmHg, and no history of ocular or systemic corticosteroid use. The inclusion criteria for the HM subjects were as follows: a BCVA of ≥ 20/40, a spherical refractive error more negative than −6 diopters, and central fixation sufficiently stable to perform image capture. Subjects with keratoconus, previous corneal lesions and prior surgery in the cornea, severe cataracts, glaucoma or posterior abnormalities, such as choroidal neovascularization, retinoschisis, retinal detachment or macular holes, were excluded.

### ET and CT measurement

**Figure 1:**
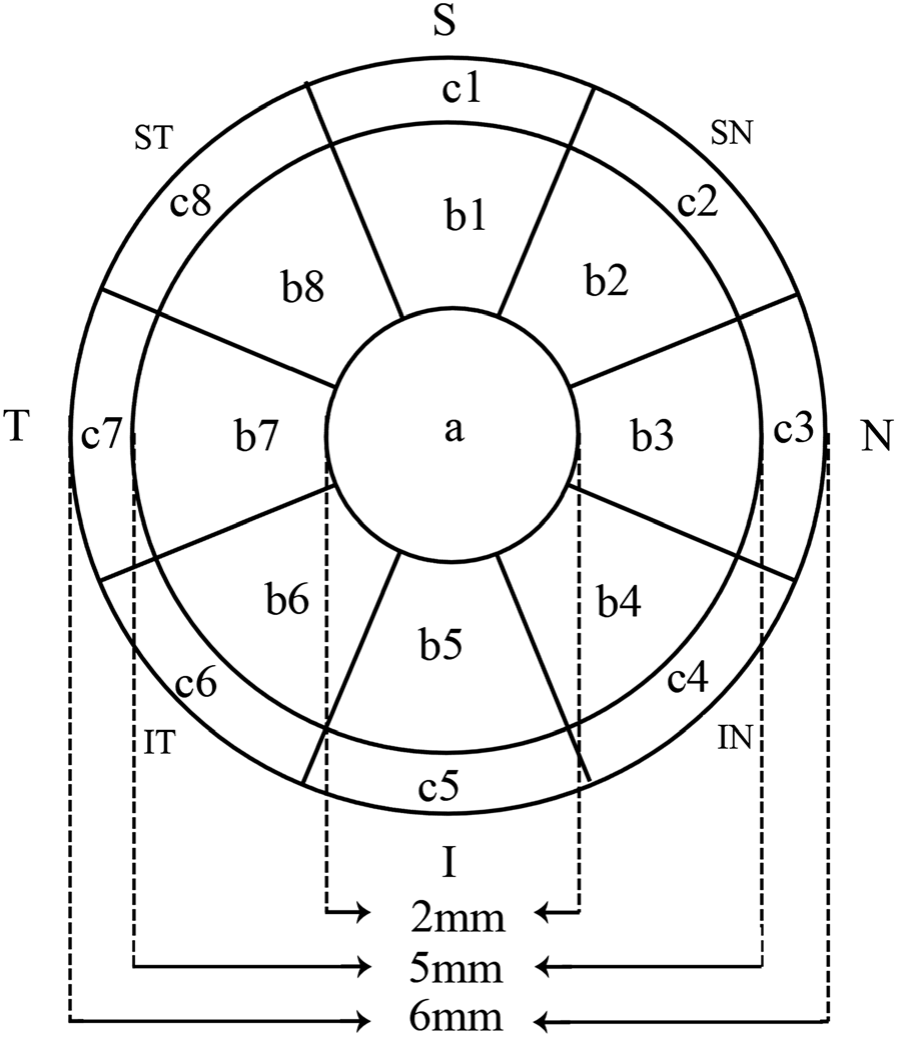
Pachymetry map of the corneal and epithelial thicknesses of 17 regions of the central 6 mm. Temporal (T); nasal (N); superior (S); inferior (I).

An Optovue RTVue (Optovue Inc., Fremont, CA, USA) and a supplemental cornea-anterior module (CAM) attachment were used for the epithelial and corneal pachymetry scans. Several studies have shown that RTVue has excellent repeatability and reproducibility in both CT and ET measurements [8,9]. The RTVue scans an area of 6 × 6 mm in the central cornea with a depth resolution approximately 5 μm. The light source of the system uses super luminescent diodes with wavelengths of 840 nm. Eight radial line scans with 1024 A-scans each for the corneal and epithelial pachymetry maps are used to demonstrate the average CT and ET in each of the 17 regions as described in our previous research [10]. These regions (Figure 1) are the center (1 mm radius, area a), the inner ring (2.5 mm radius, area b), the outer ring (3 mm radius, area c), and the eight radial scan lines in eight directions (superior (1), SN (2), nasal (3), IN (4), inferior (5), IT (6), temporal (7), and ST (8)) with angles of 45° between each consecutive scan line (a, b 1–8, c 1–8)). All of the images in this study were captured by an experienced and trained technician. All of the corneal and epithelial pachymetry data were automatically calculated by the RTVue software (version 6.11.0.12). During each scan, the technician captured each cross-sectional corneal image with the light beam at the midpoint to ensure that the scan location was in the center area of the cornea.

### AL measurement

A Lenstar LS 900 biometer (Haag-Streit AG, Koeniz, Switzerland) and its internal software (version 2.1.1)were used for the AL measurements. The Lenstar LS 900 biometer uses optical low-coherence reflectometry and a central wavelength of 820 μm to measure the AL to the retinal pigment epithelium [11]. During the examination, the patients were asked to fixate on the internal red light, and the device focus was based on the image of the eye on the monitor. The patients were asked to perform a complete blink to make ensure an optically smooth tear film over the cornea prior to image capture. Measurements that were contaminated by patient blinking or unstable fixation were excluded, and one non-contaminated measurement for each eye was used in the final analysis.

### Statistical analyses

The statistical analyses were performed with commercial software (SPSS ver. 13.0; SPSS Inc.). The means ± the standard deviations of each variable were assessed for both the normal and HM groups. To compare the ET, CT and AL variables measured in the normal and HM eyes, independent sample t-tests were used. The correlation coefficients were also calculated for age, gender, ET, CT and AL in each group. The significance level for all of the tests was set at 5%.

## Results

Data from one random eye of 97 normal subjects and 48 HM patients were analyzed (Table 1). There was no significant difference in age between the 2 groups. However, there were significant difference in gender, spherical error and astigmatism between the 2 groups.

**Table 1:** **Characteristics of normal subjects and subjects with high myopia**

**Characteristics**	**Normal**	**High myopia**	***P*** Value*
Patients, n	97	48	
Eyes, n	97	48	
Age (yrs)	56 ± 18	51 ± 20	0.188
Gender (male/female)	33/64	25/23	**0.037**
Spherical error (diopter)	−1.27 ± 0.64	−9.11 ± 3.63	0.000
Astigmatism (diopter)	0.89 ± 0.50	1.45 ± 0.89	0.000

*All calculated by t test, except the values in bold, which was calculated by chi-square test.

**Figure 2:**
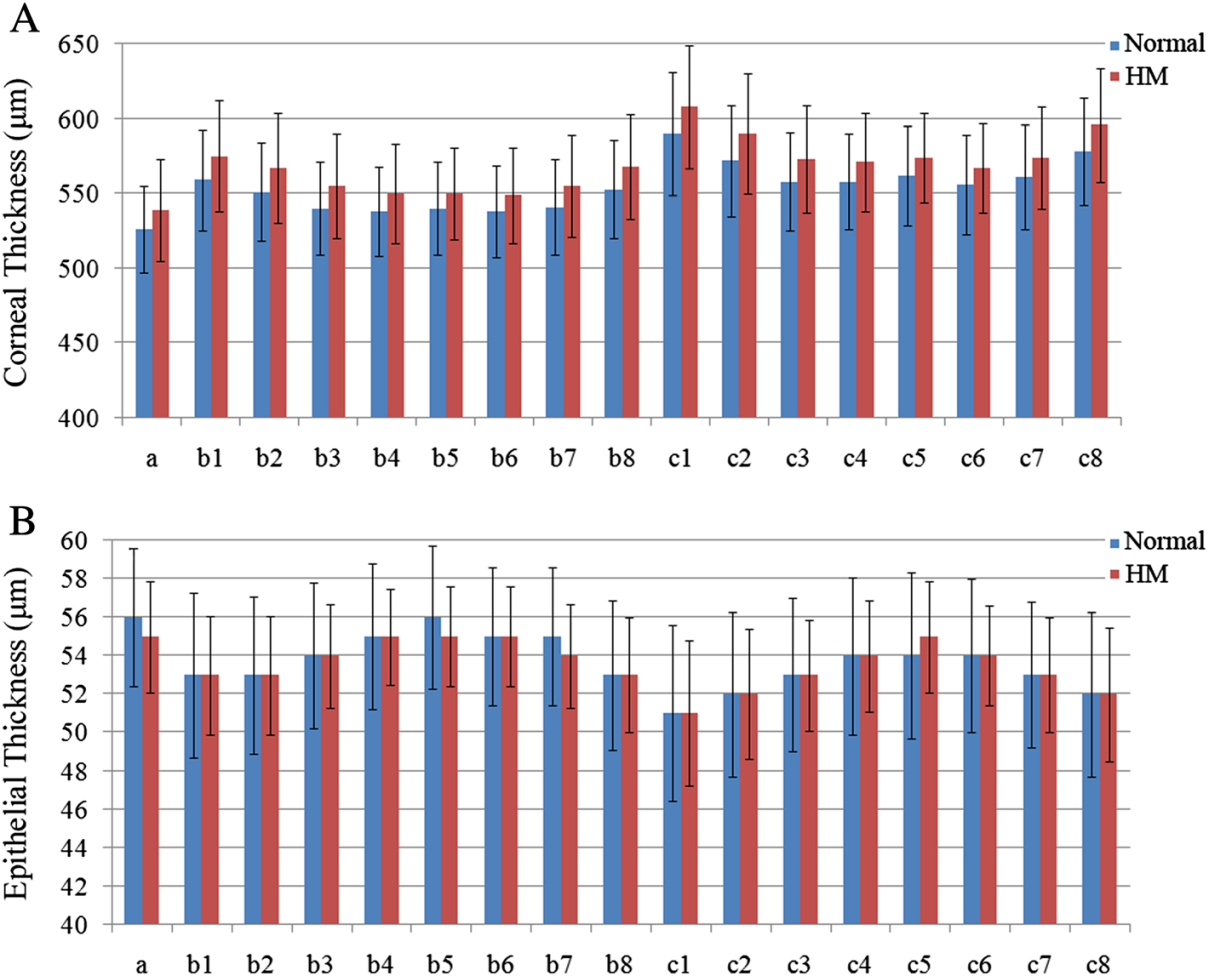
Histograms comparing the corneal thickness **(A)** and epithelial thicknesses **(B)** of the 17 regions between the normal and high myopia groups.

Significant differences in the CT and AL values were found between the normal and HM eyes, but no difference was found in the ET values (Table 2, Figure 2). Compared to the normal eyes, the ALs of the HM eyes were significantly increased by 16% (P < 0.001). In contrast to our finding of no difference in the ETs, significantly thicker CTs in the HM eyes were found in all 17 regions, and the c1, c2 and c8 regions exhibited the largest biggest differences.

**Table 2:** **Summary of the axial length, corneal thickness and epithelial thickness of normal and high myopia groups**

	**Normal**	**HM**	**Mean difference**	**P**
	**(n = 97)**	**(n = 48)**		
Axial length (mm)	23.1 ± 0.7	26.7 ± 2.1	−3.6	**0.000**
CT (μm)				
Central (a)	526 ± 29	539 ± 34	−14	**0.013**
S (b1)	559 ± 34	575 ± 37	−16	**0.011**
SN (b2)	551 ± 33	567 ± 37	−16	**0.009**
N (b3)	540 ± 31	555 ± 35	−14	**0.014**
IN (b4)	538 ± 30	550 ± 33	−12	**0.030**
I (b5)	540 ± 31	550 ± 31	−11	0.054
IT (b6)	538 ± 31	549 ± 32	−11	**0.041**
T (b7)	541 ± 32	555 ± 34	−13	**0.023**
ST (b8)	553 ± 33	568 ± 35	−15	**0.013**
S (c1)	590 ± 41	608 ± 41	−18	**0.014**
SN (c2)	572 ± 37	590 ± 40	−18	**0.008**
N (c3)	558 ± 33	573 ± 36	−15	**0.016**
IN (c4)	558 ± 32	571 ± 33	−13	**0.023**
I (c5)	562 ± 33	574 ± 30	−12	**0.041**
IT (c6)	556 ± 33	567 ± 30	−11	**0.046**
T (c7)	561 ± 35	574 ± 34	−13	**0.034**
ST (c8)	578 ± 36	596 ± 38	−18	**0.008**
ET (μm)				
Central (a)	56 ± 3.6	55 ± 2.9	0.7	0.214
S (b1)	53 ± 4.3	53 ± 3.1	0.3	0.677
SN (b2)	53 ± 4.1	53 ± 3.1	0.1	0.870
N (b3)	54 ± 3.8	54 ± 2.7	−0.2	0.763
IN (b4)	55 ± 3.8	55 ± 2.5	0.1	0.803
I (b5)	56 ± 3.7	55 ± 2.6	−0.004	0.994
IT (b6)	55 ± 3.6	55 ± 2.6	0.1	0.891
T (b7)	55 ± 3.6	54 ± 2.7	0.2	0.746
ST (b8)	53 ± 3.9	53 ± 3.0	0.3	0.656
S (c1)	51 ± 4.6	51 ± 3.8	−0.8	0.327
SN (c2)	52 ± 4.3	52 ± 3.4	−1.0	0.154
N (c3)	53 ± 4.0	53 ± 2.9	−0.3	0.571
IN (c4)	54 ± 4.1	54 ± 2.9	−0.5	0.437
I (c5)	54 ± 4.3	55 ± 2.9	−0.8	0.182
IT (c6)	54 ± 4.0	54 ± 2.6	−0.3	0.588
T (c7)	53 ± 3.8	53 ± 3.0	−0.2	0.754
ST (c8)	52 ± 4.3	52 ± 3.5	−0.4	0.614

CT = corneal thickness; ET = epithelial thickness; HM = high myopia; I = inferior; N = nasal; S = superior; T = temporal.

Statistically significant differences (P < 0.05) are bold faced.

Age was negatively associated with AL but not CCT and CET in normal group. In the HM group, age was not associated with CCT , AL or CET. In the normal group, gender has significant correlation with CET but no CCT and AL; in the HM group, gender was correlated with CCT and AL but not with CET (Table 3).

**Table 3:** **Correlation coefficient matrix for the associations of gender, age with corneal thickness, epithelial thickness, and axial length in normal and high myopia groups**

	**Normal (n = 97)**		**HM (n = 48)**	
	**Gender**	**Age**	**Gender**	**Age**
AL (mm)	−0.153 (0.134)	**−0.245 (0.016)**	**−0.310 (0.032)**	0.210 (0.151)
CCT (μm)	−0.113 (0.269)	0.077 (0.453)	**−0.411 (0.004)**	0.031 (0.832)
CET (μm)	**−0.302 (0.003)**	−0.145 (0.157)	0.056 (0.704)	−0.259 (0.076)

The table cells display the correlation coefficients (P-values are based on Pearson correlation analyses).

CCT = central corneal thickness; CET = central epithelial thickness; HM = high myopia; I = inferior; N = nasal; S = superior; T = temporal.

Statistically significant differences (P < 0.05) are bold faced.

## Discussion

Due to the longer eyeballs of the HM subjects, both the anterior and posterior segments exhibited some abnormalities [6,12]. In this study, we evaluated the correlations between age, gender, CT, ET and AL in normal and HM groups. We also investigated the differences in the CTs, ETs, ALs between the two groups. Similar to the scatter plot showing a strong association between higher myopia and longer ALs shown in the study by Kubo et al., significantly longer ALs were found in the HM group than in the normal group in the present study [13].

As the first cellular layer of the human cornea, the corneal epithelium plays an important role in the evaluation of corneal remodeling after refractive surgery [14]. Several technologies can measure ET, such as confocal microscopy, very-high frequency ultrasound, time-domain optical coherence tomography (TD-OCT) and spectral-domain optical coherence tomography (SD-OCT) [15-18]. Reinstein et al. reported CET of 53.4 ± 4.6 μm in normal corneas using very-high frequency ultrasound [16]. The measurements of Reinstein et al., which did not include pre-corneal tear film thickness, were thinner than the CET of 56 ± 3.6 μm of the normal eyes found in the present study. Tear film thickness measurements were included in our study, and may account for this difference. Previous studies have reported varying CET values for normal corneas using TD/SD-OCT, even when the same brand of OCT device was used [17-19]. These fluctuations may be attributable to different races, ages and sample sizes.

Regarding the CCT measurements, our results are very similar to those of Wong et al. who examined a sample of normal Hong Kong Chinese people using SD-OCT [20]. Compared to our previous research that has used the Galilei Scheimpflug system, OCT produced thinner CCT values [21,22]. This difference may be attributable to differences in the measuring techniques.

This study revealed significantly thicker CTs in 16 regions of the central 6 mm in the HM group. This result is in agreement with that of Kunert et al. who studied an Indian population [7]. However, this result is different from the thinner or unaltered CTs that have been reported for HM samples in previous studies [2,23]. There are several possible reasons for this difference. First, we assume that there are different stages of the changes of CT in HM; e.g., early, middle, and advanced stages. There might be different CTs associated with each stage, and research into these myopic stages should be performed. Second, CT exhibits a 24-hour fluctuation, which might have influenced the final measurement values for the statistical analyses of different studies due to differences in measurement times [24]. Third, the refractive range for HM, which may influence the CT, were different in each study. In contrast to the thicker CTs of the HM eyes, no differences in ET between the two groups were identified in any of the 17 sub-regions, which indicates that the changes in CT were primarily attributable to the corneal stroma.

In the present study, age was negatively associated with AL in the normal group, and this finding is similar to the finding of an increase in AL with decreasing age at the time of cataract surgery of the research of Tuft et al. [25]. In contrast to AL, CCT was found to remain constant with age at the resolution available using the SD-OCT in both the normal and HM groups, which exhibited results that were similar to those of previous studies that have used the Scheimpflug system and TD-OCT [22,26]. The influence of gender on CET in normal group and CCT, AL in HM group may attribute to the endocrine differences between man and women. Previous research revealed that gonadal hormones may affect ocular tissue growth by genomic or nongenomic pathway [27,28].

The current study has some limitations. First, the normal participants were not gender matched to the HM group. Second, the potential for segmentation errors by the automated software, the drift of the measurements that can be caused by instrument vibration during operation and the signal instability should be considered. Third, additional studies with larger HM samples that include multiple races and more even gender distributions are also needed. However, The corneal biometric parameters changing in HM of this study may be an important indicator for refractive surgery choosing or designing.

## Conclusions

In this report, the CTs of the HM group were significantly thicker, particularly in the c1, c2 and c8 subregions. Age and gender should be noticed for AL, CT and ET in both normal and HM group.
